# Vulnerability to violence against women or girls during COVID-19 in Uganda

**DOI:** 10.1186/s12889-022-14951-7

**Published:** 2023-01-05

**Authors:** Paul Bukuluki, Peter Kisaakye, Grace Bulenzi-Gulere, Beatrice Mulindwa, Dan Bazira, Evelyn Letiyo, Hellen Nviiri Laetitia Namirembe, Isabella Schmidt, Pamela Nabukhonzo Kakande, Simon Nissling

**Affiliations:** 1 Department of Social Work and Social Administration, School of Social Sciences, Makerere University, Kampala, Uganda; 2 Department of Population Studies, School of Statistics and Planning, Makerere University, Kampala, Uganda; 3 Gender Statistics, UN Women, Kampala, Uganda; 4 Access to Justice, UN Women, Kampala, Uganda; 5 Ending Violence Against Women, UN Women, Kampala, Uganda; 6 Directorate of Population and Social Statistics, Uganda Bureau of Statistics, Kampala, Uganda; 7 Regional Statistics, UN Women, Nairobi, Kenya

**Keywords:** Vulnerability, Gender-based violence, Violence against women or girls, COVID-19, Uganda

## Abstract

At the height of the COVID-19 pandemic, gender-based violence (GBV) was reported to have increased worldwide. We build on existing literature to examine the factors that increased vulnerability to GBV during the COVID-19 pandemic in Uganda. We use data from the Rapid Gender Assessment (RGA) survey that was conducted during COVID-19, which was designed to provide information to guide policymaking and offer appropriate interventions that address the needs of people in Uganda during the pandemic. The results show that the following respondents are more likely to experience increased risk and vulnerability to gender-based violence: those with primary level of education (OR = 1.49; 95% CI = 1.10–2.01), those who received information about GBV (OR = 1.30; 95% CI = 1.08–1.57), and those who needed help or medical support as a prevention measure against GBV (OR = 1.29; 95% CI = 1.04–1.61). However, respondents who would need financial support to prevent GBV were less likely to experience increased GBV (OR = 0.83; 95% CI = 0.70–0.98). Our results align with evidence from other studies that risk and vulnerability to GBV in Uganda increased since the onset of COVID-19. The findings provide an understanding of the interrelationship between GBV and COVID-19,which can help with designing GBV preventive measures, particularly during pandemics among those most at-risk.

## Introduction

At the height of the COVID-19 pandemic, gender-based violence (GBV) or violence against women or girls (VAWG) was reported to have increased globally [1–4];about 31 million cases of GBV were reported in the first six months of the COVID-19 pandemic [5]. By definition, GBV is any form of violence against a person based on sex or gender [6].

GBV is a human rights violation with far-reaching negative consequences on survivors, their families and children [1]. Moreover, GBV has been declared as a global pandemic – with negative consequences on health and wellbeing of individuals [2]. In Uganda, an increase in GBV during COVID-19 pandemic due to lockdowns or restrictions in movement was reported [7]. A recent study in Uganda that used cross-sectional data of 1,726 participants from the International Citizens Project (ICP) reported that about 8% of participants experienced a form of violence during COVID-19 [7].

Women or girls are more vulnerable to experiencing GBV from their partners [5]. Stress from economic hardships to provide for the family and the effects of COVID-19 during lockdowns further aggravated GBV [8]. Other factors reported in the literature for increased GBV include loss of a job [9], financial dependency [10], overcrowding [11], and difficulty to adjust to a new lockdown circumstance. Women endured abusive partners in confined environments such as their homes – places deemed safe for all [12]. Moreover, lockdowns and other control measures had a negative effect on social, mental, and psychological wellbeing [5, 13, 14], yet such measures neglected GBV prevention services [15]. Moreover, previous research shows that women may be less likely to report perpetrators of GBV if they live with their abusers [16].

The literature points to varied factors associated with GBV during COVID-19. Previous research in Uganda has reported being male or having an inability to access essential services during COVID-19 as contributing factors to GBV [7]. Analysis of data from Twitter reveals coercive control and physical aggression as major forms of violence during COVID-19, but alcohol abuse and financial difficulties as contributing risk factors [17]. Analysis of qualitative data from 12 countries pointed to alcohol abuse, school closures, and limited access to social and health services as risk factors for GBV during COVID-19 [18]. Other factors associated with GBV during COVID-19 are age difference between partners (more than four years) [19] and patriarchy [20].

GBV is a form of gender inequality that threatens sustainable development [21]. This paper seeks to enrich the existing literature by examining the factors that increased vulnerability to GBV during COVID-19 pandemic. Understanding the interrelationship between the two pandemics (GBV and COVID-19) can help with designing GBV preventive measures, particularly during pandemics among the most at-risk [22], but also protect marginalized women and girls [6].

### The context

In Uganda, VAWG remains widespread [23]. According to the recent 2016 Uganda Demographic and Health Survey (UDHS) report, 51% of adolescents (15–19 years) have experienced physical violence since age 15 [24]. About two out of ten women (22%) reported to have experienced physical violence in the last 12 months preceding the survey. 22% of women aged 15–49 years reported to have experienced sexual violence since the age 15. 13% experienced sexual violence in the last 12 months preceding survey [24]. According to the 2016 UDHS results, experience of violence is more likely to occur as women age; among divorced, separated, or widowed women; employed women; women living with a disability; those living in rural areas; uneducated women; or women living in households with low socio-economic status. In Uganda, the first COVID-19 case was identified in March 2020 [25]. Since then, the Government of Uganda introduced lockdown/stay-at-home orders and social distancing measures in a bid to stop its spread. However, the stay-at-home orders are believed to have rather increased occurrence of violence at home due to economic hardship, decline in social support structures, and overcrowding among contributing factors [26–30]. In Uganda, COVID-19 lockdowns were associated with limited access to GBV prevention services and information [31, 32], exacerbating the occurrence of violence.

## Data and methods

### Source of data and study setting

The data used in this study come from the Rapid Gender Assessment (RGA) survey that was conducted during COVID-19. The RGA data were collected by the Uganda Bureau of Statistics. The survey was designed to provide information to guide policymaking and offer appropriate interventions that address the needs of people in Uganda during COVID-19. The RGA survey was conducted in all the four regions of the country (Central, Eastern, Northern, and Western) – Acholi, Ankole, Buganda, Bukedi, Bunyoro, Busoga, Elgon, Kampala, Karamoja, Kigezi, Lango, Teso, Tooro, and West-Nile.

### Sample size and data collection

Information was collected from men and women who were 18 years or older using a semi-structured questionnaire. A sample of 3,001 respondents was systematically selected using random stratified sampling method. For representativeness, respondents were stratified by age, sex, and region. Respondents that were included in the study were selected from the GEOPOLL database. Interviews were conducted in English and in local languages (Luganda, Lugisu, Luo, Ateso-Karimajong, Runyankore-Rukiga, Lusoga, Lugbara, and Runyoro-Rutoro) by well-trained interviewers. Data collection took place between 31 October and 30 November 2020. On average, the total duration for interviews was between 45 min and 1 h. Computer-assisted telephonic interview (CATI) technology was used to collect data. Given the fact that data collection took place during COVID-19, Standard Operating Procedures (SOPs) were followed as guided by the World Health Organization (WHO) [33]. No incentive was provided to participants of the survey. Interviewers made a total of three attempts on different dates/times to conduct the interview before concluding that the selected person could not be traced.

### Measurement of variables

#### Dependent variable

The dependent variable is binary because responses to the question were either “Yes” or “No”. Respondents were asked whether they personally experienced increased risk and vulnerability toward GBV during COVID-19.

#### Independent variables

Variables include the sex of the respondents (female or male), age (18–27, 28–37, and 38+), current marital status (currently married or not currently married), type of residence (urban or rural), level of education (no education, primary, secondary, vocational, or university), and current employment status (yes or no). Respondents were asked whether they ever received information about GBV or sexual and reproductive health and rights (SRHR) during COVID-19 (yes or no) from radio, television, neighbor, online/social media, friend, community activist/volunteer, or family member. Respondents provided information on people’s needs (things they would need) to prevent GBV: financial help, help on how to report, crime prevention, psychosocial support, legal support, help on insurance claim, medical support, police support, moral support, judge prosecutor, and victim protection. A response to each of the questions was either yes or no.

### Data analysis

Data analysis was performed using the Stata software version 15 [34]. We fitted a simple binary logistic regression model using a step-wise approach to identify the factors that were associated with increased risk and vulnerability to GBV during COVID-19.

## Results

### Respondent’s characteristics

Table 1 show the distribution of respondents in the sample. Slightly more than half of respondents (51%) were male, living in urban areas (56%), or currently married (58%). One-third of respondents (33%) were 38-years-old and above. About 39% of respondents had secondary level education. Most respondents (66%) were currently engaged in employment for payment (at the time of the survey). About seven out of every ten respondents reported to have received some information about GBV prevention during COVID-19, and 63% of respondents had previously received some information about SRHR during COVID-19. About three in four respondents (76%) felt that GBV in Uganda had increased since the onset of COVID-19.

**Table 1:** Socio-demographic characteristics of respondents (*N*=3001)

Variable	Frequency	Percent
Sex		
Female	1465	48.8
Male	1536	51.2
Age (Mean = 35.4)		
18-27	1041	34.7
28-37	960	32.0
38+	1000	33.3
Current marital status		
Currently married	1753	58.4
Not currently married	1248	41.6
Type of residence		
Rural	1309	43.6
Urban	1692	56.4
Level of education		
No education	103	3.4
Primary	305	10.2
Secondary	1158	38.6
Vocational	423	14.1
University	1012	33.7
Currently doing work		
No	1035	34.5
Yes	1966	65.5
Ever received any information about GBV during COVID-19		
Yes	2293	76.4
No	708	23.6
Ever received any information about SRHR during COVID-19		
Yes	1892	63.1
No	1109	36.9
Perception on whether GBV increased during COVID-19 in Uganda		
Increased	2294	76.4
Decreased	290	9.7
Remained the same	249	8.3
Do not know	168	5.6

**Fig. 1:**
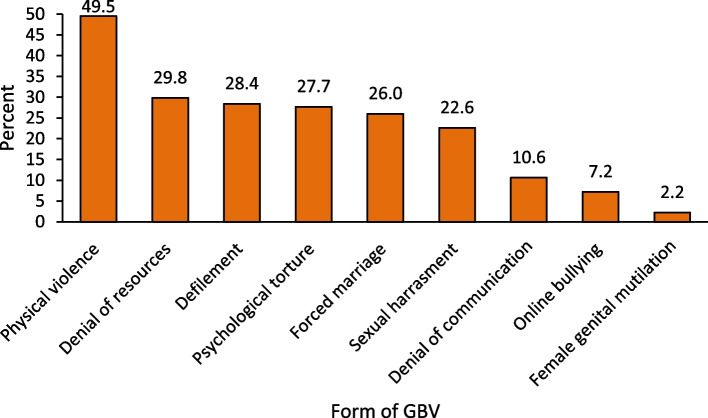
Participant experiences of different forms of gender-based violence in the last 6 months (*N* = 1417)

Figure 1 shows the different forms of GBV that were experienced in the last 6 months preceding the survey by respondents in the study. Respondents were asked the different forms of GBV they experienced the most recently. Physical violence was the most experienced form of GBV: about half (49%) of respondents experienced physical violence in the last 6 months preceding the survey. About one-third (30%) were denied resources while 28% were defiled or psychologically tortured. The least reported form of GBV experienced was female genital mutilation (2%).

### Reported information needed to prevent GBV during COVID-19

**Fig. 2:**
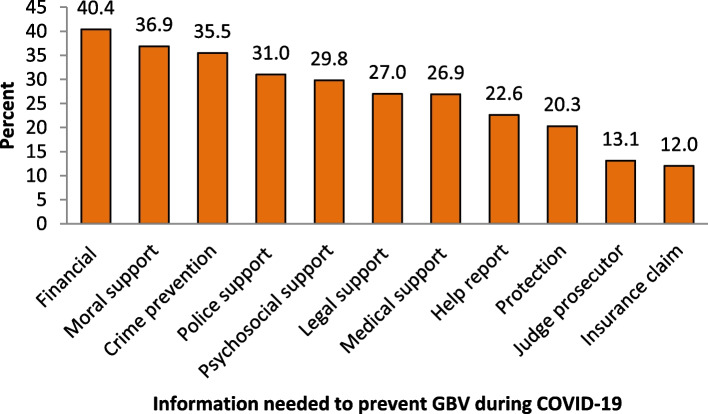
Reported information required to prevent GBV during COVID-19 (*N* = 3001)

Figure 2 shows the different types of reported help and information that would be needed to prevent GBV during COVID-19. Respondents were asked the types of information, advice, or support they would need to prevent GBV, VAWG, or other harmful practices during the COVID-19 pandemic. Financial assistance was reported by most respondents (40%), followed by moral support (37%). Figure 2 shows that the least proportion of respondents would need support and information from the judge prosecutor (13%) and for insurance claims (12%).

### Relationship between selected background variables and vulnerability to GBV during COVID-19

The results in Table 2 show that most respondents experienced greater risks and vulnerability to GBV during COVID-19 (66%). Results indicate that respondents were significantly different by the level of education (*p* < 0.05), whether they received information about GBV prevention (*p* < 0.01), SRHR (*p* < 0.05), and the risk of GBV during COVID-19. Respondents were also significantly different by the risk to GBV and the level of support needed such as financial (*p* < 0.01), help in preventing the occurrence of GBV (*p* < 0.05), psychosocial support (*p* < 0.01), legal help (*p* < 0.05), insurance claim (*p* < 0.05), police help (*p* < 0.01), moral support (*p* < 0.05), help on judge prosecutor (*p* < 0.01) and support on victim protection (*p* < 0.05).

**Table 2:** Relationship between selected variables and greater risks and vulnerability to GBV during COVID-19

Variable	Experienced greater risks and vulnerability to violence during COVID-19		Chi-square (*p*-value)	Total (N)
	Yes	No		
Sex			0.298 (0.585)	
Female	65.5	34.5		1465
Male	66.4	33.6		1536
Age			0.665 (0.717)	
18–27	65.2	34.8		1041
28–37	65.7	34.3		960
38+	66.9	33.1		1000
Current marital status			1.039 (0.308)	
Currently married	66.7	33.3		1759
Not currently married	64.9	35.1		1242
Type of residence			3.413 (0.065)	
Rural	67.8	32.2		1309
Urban	64.5	35.5		1692
Level of education			11.218 (0.024)*	
No education	70.9	29.1		103
Primary	73.4	26.6		305
Secondary	65.3	34.7		1158
Vocational	66.4	33.6		423
University	63.7	36.3		1012
Currently doing work			0.471 (0.492)	
No	66.8	33.2		1035
Yes	65.5	34.5		1966
Ever received any information about GBV during COVID-19			6.869 (0.009)**	
No	61.9	38.1		708
Yes	67.2	32.8		2293
Ever received any information about SRHR during COVID-19			5.110 (0.024)*	
No	63.4	36.6		1109
Yes	67.4	32.6		1892
Need financial support to prevent GBV during COVID-19			7.084 (0.008)**	
No	67.8	32.2		1788
Yes	63.2	36.8		1213
Need help to report GBV incident to prevent GBV during COVID-19			5.782 (0.016)*	
No	62.1	37.9		678
Yes	67.1	32.9		2323
Need crime prevention skills to prevent GBV during COVID-19			3.039 (0.081)	
No	67.1	32.9		1937
Yes	63.9	36.1		1064
Need psychosocial support to prevent GBV during COVID-19			6.903 (0.009)**	
No	67.4	32.6		2106
Yes	62.5	37.5		895
Need legal support to prevent GBV during COVID-19			5.013 (0.025)*	
No	67.1	32.9		2190
Yes	62.8	37.2		811
Need help on insurance claim to prevent GBV during COVID-19			4.081 (0.043)*	
No	66.6	33.4		2640
Yes	61.2	38.8		361
Need help on medical support to prevent GBV during COVID-19			0.320 (0.571)	
No	66.2	33.8		2195
Yes	65.1	34.9		806
Need police support to prevent GBV during COVID-19			8.638 (0.003)**	
No	67.6	32.3		2071
Yes	62.2	37.9		930
Need moral support to prevent GBV during COVID-19			4.197 (0.040)*	
No	67.3	32.7		1893
Yes	63.6	36.4		1108
Need help on judge prosecutor to prevent GBV during COVID-19			8.016 (0.005)**	
No	66.9	33.1		2607
Yes	59.6	40.4		394
Need help to protect victim from GBV during COVID-19			4.541 (0.033)*	
No	66.9	33.1		2391
Yes	62.3	37.7		610
Perception on whether GBV increased during COVID-19 in Uganda			0.391 (0.942)	
Increased	66.2	33.8		2294
Decreased	65.5	34.5		290
Remained the same	65.1	34.9		249
Do not know	64.3	35.7		168
Total (%)	65.9	34.1		100
Total (N)	1979	1022		3001

Note: *=*p* < 0.05; **=*p* < 0.01

### Factors associated with increased risk and vulnerability to GBV during COVID-19

The results presented in Table 3 show that respondents with primary level of education were more likely (OR = 1.49; 95% CI = 1.10–2.01) to experience increased risk and vulnerability to GBV during COVID-19 than their counterparts with university level of education. Respondents who ever received information about GBV were more likely to experience increased risk and vulnerability to GBV (OR = 1.30; 95% CI = 1.08–1.57) than respondents who did not receive any information. Respondents who would need financial support to prevent GBV were less likely to experience increased GBV (OR = 0.83; 95% CI = 0.70–0.98) than their counterparts. The results in Table 3 show that respondents who needed help on medical support as a preventing measure against GBV were more likely to experience increased risk and vulnerability (OR = 1.29; 95% CI = 1.04–1.61) than respondents who said no.

**Table 3:** Factors associated with increased risk and vulnerability to GBV during COVID-19

Variable	Odds Ratio (95%CI)
Sex (RC= Male)	
Female	0.97 (0.83-1.14)
Age (RC=18-27)	
28-37	0.98 (0.80-1.20)
38+	0.96 (0.78-1.19)
Current marital status (RC=Not currently married)	
Currently married	1.04 (0.87-1.23)
Type of residence (RC=Rural)	
Urban	0.91 (0.77-1.06)
Level of education (RC=University)	
No education	1.34 (0.85-2.12)
Primary	1.49 (1.10-2.01)**
Secondary	1.05 (0.87-1.26)
Vocational	1.15 (0.90-1.47)
Currently doing work (RC=Yes)	
No	1.05 (0.89-1.25)
Ever received any information about GBV during COVID-19 (RC= No)	
Yes	1.30 (1.08-1.57)**
Ever received any information about SRHR during COVID-19 (RC= Yes)	
No	0.85 (0.72-0.99)
Need financial support to prevent GBV during COVID-19 (RC=No)	
Yes	0.83 (0.70-0.98)*
Need help to report GBV incident to prevent GBV during COVID-19 (RC=No)	
Yes	0.94 (0.76-1.17)
Need crime prevention skills to prevent GBV during COVID-19 (RC= No)	
Yes	0.96 (0.80-1.14)
Need psychosocial support to prevent GBV during COVID-19 (RC=No)	
Yes	0.89 (0.73-1.08)
Need legal support to prevent GBV during COVID-19 (RC=No)	
Yes	0.93 (0.77-1.14)
Need help on insurance claim to prevent GBV during COVID-19 (RC=No)	
Yes	0.99 (0.75-1.33)
Need help on medical support to prevent GBV during COVID-19 (RC=No)	
Yes	1.29 (1.04-1.61)*
Need police support to prevent GBV during COVID-19 (RC=No)	
Yes	0.84 (0.70-1.01)
Need moral support to prevent GBV during COVID-19 (RC=No)	
Yes	0.93 (0.78-1.11)
Need help on judge prosecutor to prevent GBV during COVID-19 (RC=No)	
Yes	0.86 (0.66-1.13)
Need help to protect victim from GBV during COVID-19 (RC=No)	
Yes	0.96 (0.77-1.21)
Perception on whether GBV increased during COVID-19 in Uganda (RC=No)	
Yes	0.99 (0.76-1.30)
Remained the same	0.97 (0.68-1.40)
Do not know	0.92 (0.61-1.39)
Constant	1.99 (1.46-2.70)***

Note: *=*p* < 0.05; **=*p* < 0.01*p* < 0.001

## Discussion

In Uganda during the COVID-19 pandemic, the most prevalent form of GBV experienced was physical violence, but significant proportions of respondents experienced sexual violence, denial of resources, and psychological violence. This is in line with other studies that revealed that coercive control and physical aggression are major forms of violence experienced during COVID-19 [17]. It also aligns with the evidence from other studies in Uganda and in other low-income settings that show increase in sexual, emotional, and economic (denial of resources) violence [1–5, 10].

Our study reveals several factors associated with increased risk and vulnerability to GBV during COVID-19. Socio-economic status particularly linked to low education achievement (primary education) and the need for assistance to access health care was associated with higher likelihood to experience increased risk and vulnerability to GBV. These results are similar to other studies that show that loss of a job [9], financial dependency [10] and difficulty to adjust to lockdown circumstances increased vulnerability to GBV during COVID-19 [5].

However, our results are quite contrary to other studies especially in respect to respondents who would need financial support to prevent GBV, being less likely to experience increased GBV. This may point to the notion that the need for financial support alone may be inadequate as a determinant of vulnerability to GBV. There could be other context specific factors that need to be explored that combine with the need for financial support to influence women or girls’ vulnerability to GBV during COVID-19. Similarly, those reported to have ever received information about GBV against women or girls were more likely to experience increased risk and vulnerability to GBV. This could imply that receiving information on GBV prevention alone is a weak determinant for risk and vulnerability to GBV [35]. It suggests that the package of information, its relevance to the target population and their context may play a central role in its effectiveness in influencing risk and vulnerability to GBV [35, 36]. Our results also demonstrate that those who reported that they needed medical support as a preventive measure against GBV were more likely to experience increased risk and vulnerability to GBV. This means that the expression of need for medical support could be a proxy for other markers of risk and vulnerability to GBV that go beyond the bio-medical aspects to include structural factors that need to be taken into consideration in GBV prevention programming [37]. This finding underscores the need to offer a comprehensive package of GBV prevention services that go beyond medical (bio-medical services) to address risk factors to GBV that manifest at different levels of the social ecology—individual, family, community, and society levels [37]. This is also in line with the recommendation by the WHO for a comprehensive package of GBV prevention and response services that include health/medical, mental health and psychosocial support, protection, legal/justice or legal aid, and counselling [38].

Overall, results point to the reality that women or girls are more vulnerable to experiencing GBV from their partners but also non-intimate partners during times of pandemics. These results cannot be properly understood without making reference to the socio-cultural context. For example, in our study setting where patriarchal systems exist [20], some scholars have described this as contributing to hegemonic masculinity [39] or toxic masculinities – that are generally critical to contest stern adherence to masculinized gender norms [40] – with the goal of transforming these harmful norms into positive masculinities [41]. In this case, it has been argued that hegemonic masculinity describes how gender, power and oppression are embodied in relationships between men and women [39]. Therefore, women’s vulnerability and experience of GBV during COVID-19 is a continuation of their already existing risks to GBV embedded within the structures of patriarchy that provide a fertile ground for hegemonic masculinity to encompass an explicit strategy for the subordination of women [39, 42, 43]. This contributes to normalizing hierarchical gender relations between men and women, to the extent of such relations being perceived as normal or even legitimate [44]. It is from this perspective that we argue that the increase in vulnerability and experience of GBV during pandemics such as COVID-19 in Uganda and similar socio-cultural settings must be understood in the overall context of structural factors related to patriarchy and their implications for constraining more gender equal norms and practices.

Similarly, the literature on social and gender norms as well as the social norms theory helps to re-affirm our findings and arguments. Social norms theory [45] is instructive in our understanding of how to use findings from this study in recommending for improved implementation of policy and practice related to preventing GBV both in the context of global pandemics such as COVID-19 – more so, when this is happening in the context of patriarchal social norms and power hierarchies between men and women. As argued by Mackie et al. (2015) and other scholars [46], social norms theory facilitates an understanding of how behavior and practices of individuals are influenced by social and/or gender norms at the community level and the importance of social sanctions in reinforcing adherence or normalization of such behavior [47].

Based on these results, we argue that the vulnerability and experience of GBV during COVID-19 should be understood in the socio-cultural context associated with acceptance of hegemonic masculinity and normalization of toxic masculinities that are intentional at subordinating women. These operate in the context where harmful social norms that tolerate and reinforce GBV practices are commonplace. This implies that interventions intending to prevent occurrence or reducing of vulnerability of women and girls to GBV in patriarchal settings should consider adopting social norms change strategies even long before pandemics such as COVID-19 occur. This is not to say that addressing norms is the panacea to prevention of GBV; indeed, prevention of GBV during pandemics such as COVID-19 should have multi-thronged approaches that address norms and other structural [46] and context specific drivers of GBV during COVID-19.

### Study limitations

There are two major limitations: first, the cross-sectional nature of the data used in the study implies that the results reported in this study may not be free from recall bias, as respondents fail to recall events that happened at the exact times events occurred. Second, given that this was a telephonic survey, there is a possibility of inadvertently excluding respondents (mainly in rural areas) without functional phones or with poor network coverage, making generalizability difficult.

## Conclusion

Overall, our results demonstrate that vulnerability and experience of GBV increased during the COVID-19 pandemic in Uganda. Women and girls were prone to all types of violence especially physical violence, sexual violence, psychological violence, and denial of resources. Several factors are associated with vulnerability to GBV during COVID-19 including low socio-economic status, especially low education levels and the need for assistance for basic requirements such as medical support or health care. While the findings in the present study can be attributed to COVID-19, given the timing of implementation, the cross-sectional nature of the data used in the study makes it difficult to establish causality between vulnerability to GBV and COVID-19.

## Data Availability

The datasets used in this study are available from the corresponding author on reasonable request.
